# Beyond Antibiotics: Bacteriophage Therapy Against Multidrug-Resistant Pathogens in the Era of Antimicrobial Resistance

**DOI:** 10.7759/cureus.116535

**Published:** 2026-09-19

**Authors:** Benny Vijayanandh Avanigadda, Raja Krishna Venigalla, Renusri Ede, Pranathi Reddy Jetti, Venkataramana Kandi, Tarun Kumar Suvvari

**Affiliations:** 1 Internal Medicine, Andhra Medical College, Visakhapatnam, IND; 2 Medicine, Guntur Medical College, Guntur, IND; 3 Internal Medicine, NRI Medical College, Guntur, IND; 4 Internal Medicine, Gandhi Medical College, Hyderabad, IND; 5 Clinical Microbiology, Prathima Institute of Medical Sciences, Karimnagar, IND; 6 General Medicine, Rangaraya Medical College, Kakinada, IND; 7 Research, Squad Medicine and Research (SMR), Amadalavalasa, IND

**Keywords:** amr, antimicrobial resistance, bacteriophages, multi-drug resistance, phages, phage therapy

## Abstract

The escalating global health crisis of antimicrobial resistance (AMR) urgently calls for interventions that transcend the limitations of conventional antibiotics. Bacteriophages (phage therapy) is emerging as a critical therapeutic and prophylactic agent with a distinct operational role against multidrug-resistant (MDR) pathogens. The core of the phage's utility lies in three strategic advantages: high specificity, self-amplification at the site of infection, and most crucially, their unique capacity for biofilm eradication. Since up to 80% of chronic infections involve biofilms that shield bacteria from antibiotics, the ability of phages to penetrate and disperse these structures makes them invaluable against recalcitrant infections. Phages are thus positioned not merely as an alternative, but as a strategic and complementary tool alongside antibiotics, often demonstrating synergy that can resensitize resistant bacteria to previously ineffective drugs. Overcoming current regulatory and manufacturing challenges is essential to unlock the full potential of phage therapy and secure its vital role in the global fight against AMR.

## Editorial

Introduction

Antimicrobial resistance (AMR) represents one of the most pressing global health emergencies of the twenty-first century. The Global Burden of Disease Study 2019 estimated 1.27 million deaths directly attributable to bacterial AMR and 4.95 million deaths associated with resistant infections [1]. Gram-negative pathogens, such as *Acinetobacter (A.) baumannii, Klebsiella (K.) pneumoniae, and Pseudomonas (P.) aeruginosa,* account for a disproportionate share of carbapenem-resistant infections worldwide [1,2]. The antibiotic development pipeline has failed to keep pace with resistance evolution. Economic disincentives, rapid emergence of resistance, and limited innovation have resulted in a critical therapeutic gap [3]. In this context, bacteriophage therapy has re-emerged as a biologically adaptive strategy capable of addressing multidrug-resistant (MDR) infections through mechanisms fundamentally distinct from conventional antibiotics.

Why are bacteriophages strategically advantageous in MDR infections?

Bacteriophages, or phages, are viruses that selectively infect and replicate within bacteria [4]. Therapeutic phage preparations generally employ strictly lytic phages, which attach to susceptible bacterial cells, replicate intracellularly, and ultimately cause bacterial lysis and release of progeny phages. Their host specificity enables targeting of pathogenic strains while potentially exerting less collateral pressure on the surrounding microbiota than broad-spectrum antibiotics [4,5]. Importantly, phages can be administered alone or in combination with antibiotics and may be delivered systemically or locally, depending on the site and characteristics of infection. Contemporary phage therapy increasingly incorporates genomic characterization, susceptibility testing, phage cocktails, and individualized phage selection, allowing treatment to be tailored to the infecting strain [4,5]. This combination of biological specificity, adaptability, and potential antibiotic synergy has renewed interest in phages as an adjunctive strategy against otherwise difficult-to-treat MDR infections.

Unlike broad-spectrum antibiotics, lytic bacteriophages typically exhibit narrow host ranges determined by specific bacterial surface receptors, allowing targeted elimination of susceptible pathogens while potentially exerting less disruption of the commensal microbiota. This specificity may be particularly advantageous in settings where antibiotic-associated dysbiosis and opportunistic infections, including *Clostridioides (C.) difficile* infection, are important concerns [4]. Phages also possess a distinctive replicative property: following infection of susceptible bacteria, they can undergo successive rounds of replication at the site of infection, potentially increasing phage concentrations locally while the target bacterial population remains available. This dynamic pharmacology differs fundamentally from conventional antibiotics, whose concentrations are primarily determined by administration and host pharmacokinetics [6]. Another potential advantage is activity against biofilm-associated bacteria. Biofilms are implicated in a substantial proportion of chronic and device-associated infections, including prosthetic joint infections, chronic wounds, catheter-associated infections, and respiratory infections in cystic fibrosis [7]. Certain phages and phage-derived enzymes, particularly depolymerases, can disrupt components of the extracellular polymeric matrix and facilitate access to bacteria embedded within biofilms, thereby complementing antibiotic activity [8]. Finally, the evolutionary interaction between phages and bacteria may provide opportunities for therapeutic synergy with antibiotics. Bacterial adaptations that reduce phage susceptibility, such as alterations in surface receptors or membrane-associated structures, can sometimes impose fitness costs or alter antibiotic susceptibility, resulting in phenotypes that may be exploitable through sequential or combined phage-antibiotic treatment [9,10].

What clinical evidence supports phage therapy?

Although large randomized phase III trials remain limited, emerging clinical evidence supports the feasibility and generally favorable tolerability of bacteriophage therapy for otherwise difficult-to-treat infections. Current clinical evidence is derived predominantly from compassionate-use reports, early-phase studies, and a limited number of randomized trials, while robust phase III evidence remains scarce. Compassionate-use reports have documented clinical improvement in refractory infections caused by *A. baumannii* and *M. abscessus*, including disseminated disease, demonstrating the potential of personalized phage cocktails when conventional options are exhausted [11,12]. Early prospective studies have further established the feasibility of intravenous, topical, and intravesical administration, with no consistent signal of major phage-related systemic toxicity [11-15]. However, efficacy remains less certain: randomized evidence has not yet demonstrated clear superiority over standard therapy or placebo, as illustrated by the PhagoBurn trial and the randomized UTI trial, while heterogeneity in phage selection, dose, route, formulation, and patient populations complicates cross-study comparisons [13]. These findings highlight a central translational challenge, i.e., moving from successful individualized rescue therapy to reproducible, adequately powered clinical trials with standardized manufacturing, potency, pharmacokinetic and immunological assessment, and predefined efficacy endpoints. Representative clinical studies highlighting the evolving evidence base for bacteriophage therapy in MDR infections are summarized in Table 1.

**Table 1:** Key clinical studies and trials evaluating bacteriophage therapy in MDR infections CF, cystic fibrosis; GMP, good manufacturing practice; IV, intravenous; IND, investigational new drug; MDR, multidrug-resistant; RCT, randomized controlled trial; UTI, urinary tract infection; TURP, transurethral resection of the prostate

Study (Year)	Design and n (sample size)	Pathogen / Condition	Intervention	Key Findings	Limitations
Schooley RT et al. (2017) [11]	Compassionate use (single-patient)	MDR Acinetobacter baumannii; disseminated necrotizing pancreatitis with septic shock	IV + percutaneous personalized phage cocktail (adjunctive to antibiotics)	Clinical recovery from near-fatal MDR sepsis; first modern US IV phage therapy case; catalyzed IND pathway	Single case; observational; concurrent antibiotics confound attribution
Dedrick RM et al. (2019) [12]	Compassionate use (single-patient)	MDR Mycobacterium abscessus; disseminated infection post-lung transplant (CF)	IV cocktail of three phages, one genetically engineered from a lysogenic phage	Marked clinical, radiographic, and microbiological improvement; first therapeutic use of engineered phages	Single case; species-specific phages; complex, resource-intensive workflow
Jault P et al. (2019) (PhagoBurn) [13]	Randomized, controlled, phase 1/2 (multicenter); n=27	Pseudomonas aeruginosa; infected burn wounds	Topical 12-phage cocktail (PP1131) vs standard silver sulfadiazine	First European RCT; safety confirmed; slower bacterial reduction than comparator, largely due to loss of phage titer during storage	Underpowered; titer degradation; limited to topical application
Petrovic Fabijan A et al (2020) [14]	Single-arm, non-comparative, first-in-human GMP trial;n=13	Severe Staphylococcus aureus infection (bacteremia, endocarditis)	IV GMP-grade three-phage cocktail (AB-SA01), twice daily × 14 days, adjunctive to antibiotics	No treatment-related adverse events over 90 days; established safety of IV GMP phage therapy in critically ill patients	No control arm; efficacy not powered as endpoint; concurrent antibiotics
Leitner L et al. (2021) [15]	Randomized, placebo-controlled, double-blind (phase 2/3);n=113	UTI uropathogens (E. coli, Enterococcus, Streptococcus, P. aeruginosa, S. aureus, Proteus) in men undergoing TURP	Intravesical Pyophage cocktail vs antibiotics vs placebo × 7 days	Well tolerated; microbiological success 18% (phage) vs 35% (antibiotics) vs 28% (placebo) — non-inferior to antibiotics but not superior to placebo	Underpowered; specific surgical population; low phage titers questioned

Translational barriers and regulatory challenges

Despite their biological promise, several barriers continue to impede the translation of phage therapy into routine clinical practice. Regulatory frameworks are evolving but remain heterogeneous, reflecting the unique characteristics of phages as self-replicating, strain-specific biological agents and the need for individualized formulations. Recent European initiatives, including harmonized quality requirements for phage medicinal products and emerging regulatory pathways, represent important steps toward standardized development and manufacture [16-18]. Phage susceptibility testing also lacks the methodological standardization of conventional antimicrobial susceptibility testing, complicating rapid and reproducible phage-patient matching. In addition, rapid identification of a suitable phage and preparation of an individualized formulation may be logistically challenging, particularly when treatment decisions are required urgently in severe infections [17-19].

Manufacturing presents additional challenges, requiring genomic characterization to exclude undesirable genes, stringent control of bacterial contaminants and endotoxin, validated potency assays, sterility, stability, and batch consistency requirements that become particularly complex when personalized cocktails must be produced rapidly [19]. Finally, phage pharmacokinetics and pharmacodynamics remain incompletely defined; tissue distribution, reticuloendothelial clearance, neutralizing antibodies, route of administration, and emergence of phage resistance can substantially influence exposure and therapeutic response [20]. Addressing these interconnected challenges will require harmonized regulatory standards, validated susceptibility and potency assays, scalable GMP manufacturing, and innovative models for individualized production and reimbursement. Without such infrastructure, the field risks remaining dependent on compelling rescue cases rather than progressing toward reproducible, evidence-based clinical use.

Is phage therapy a complement or an alternative?

Current evidence suggests that bacteriophages are best positioned as a complementary rather than replacement strategy for antimicrobial therapy, particularly in refractory MDR infections, biofilm-associated infections, and situations in which conventional options are limited [21,22]. Their potential to disrupt biofilms, act synergistically with antibiotics, and impose evolutionary trade-offs that may restore susceptibility to selected antimicrobials further expands their therapeutic relevance, although these effects require stronger clinical validation [22,23]. Phage therapy may also have a role in targeted outbreak control when the causative strain is susceptible to a defined phage preparation, but this remains an emerging application rather than an established standard of care.

The next phase of development should therefore move beyond isolated rescue cases toward precision antimicrobial medicine, integrating rapid pathogen identification, whole-genome sequencing, standardized phage susceptibility testing, and curated phage libraries to enable timely pathogen-phage matching. The recent EUCAST initiative to establish a reference methodology for phage susceptibility testing represents an important step toward this goal [19]. Artificial intelligence and machine-learning approaches for strain-level phage-host prediction may further accelerate phage selection, although their clinical utility will depend on larger, better-annotated datasets and prospective validation [24]. At the clinical level, adequately powered adaptive trials should evaluate phages in well-defined populations and determine optimal combinations with antibiotics, dosing strategies, routes of administration, and biomarkers of response. Equally important will be longitudinal assessment of phage resistance, neutralizing immunity, microbiome effects, and treatment durability. Ultimately, globally aligned regulatory standards, interoperable phage repositories, and scalable manufacturing networks will be essential to transform bacteriophages from individualized experimental interventions into a reproducible precision-antimicrobial platform for MDR infections.

Conclusion

The global escalation of multidrug resistance demands innovative strategies beyond incremental antibiotic modification. Bacteriophage therapy offers precision targeting, biofilm disruption, evolutionary adaptability, and potential synergy with existing antibiotics. While regulatory, manufacturing, and logistical challenges persist, strengthening translational infrastructure could facilitate the development of phage therapy as an important component of precision antimicrobial strategies. Further progress will depend on adequately powered clinical trials, standardized manufacturing and susceptibility testing, and greater regulatory harmonization to establish its efficacy, safety, and feasibility for broader clinical implementation.
